# Extended breastfeeding for over one year is associated with a higher likelihood of underweight and stunting among children under 3 years of age in Ethiopia, EDHS from 2000–2019

**DOI:** 10.1371/journal.pgph.0004236

**Published:** 2025-04-08

**Authors:** Maleda Tefera, Haymanot Mezmur

**Affiliations:** Haramaya University, College of Health and Medical Sciences, School of Nursing, Harar, Ethiopia; Jimma University, ETHIOPIA

## Abstract

The World Health Organization (WHO) has implemented various strategies to combat undernutrition in developing countries, with one of the most common recommendations being to breastfeed for two years or beyond. This is based on the belief that breast milk provides essential nutrition for growth and development, particularly in low-income settings. However, insufficient research explores the relationship between breastfeeding duration and undernutrition..The Ethiopia Demographic and Health Survey 2000–2019 was used to perform the study, which included 18,580, children aged three and under. A multilevel logistic regression analysis was conducted to explore the relationship between undernutrition status and independent variables, including breastfeeding duration. The strength of the association was assessed using an adjusted odds ratio (AOR) with a 95% confidence interval (CI). A P-value of less than 0.05 was considered statistically significant. The overall prevalence of undernutrition among children under 3 years of age was 51.8% (95% CI: 51.10, 52.54). Specifically, the prevalence of stunting was 42.9% (95% CI: 42.14, 43.56), followed by underweight at 30.28% (95% CI: 29.63, 30.92), and wasting at 13.84% (95% CI: 13.35, 12.04).Children breastfed into their third year of life were more likely to develop underweight (AOR: 1.55; 95% CI 1.41, 1.70) and stunting (AOR: 5.45; 95% CI 4.83, 6.15). Conversely, the likelihood of wasting decreased in children breastfeeding in their second and third year of life (AOR: 0.75; 95% CI 0.67, 0.83) and (AOR: 0.50; 95% CI 0.43, 0.60) respectively. Similarly, children who breastfed until the second year of life had a greater chance of underweight and stunting. This study highlights a significant prevalence of undernutrition among children. Children breastfed into their third year of life were more likely to experience underweight and stunting, while breastfeeding during this period was associated with a lower likelihood of wasting.

## Introduction

Nutrition plays a crucial role in shaping an individual’s growth and development. A healthy growth trajectory is indicative of sufficient nutrient intake and overall well-being. Childhood malnutrition has been associated with developmental delays, lower academic achievement, and a heightened susceptibility to chronic illnesses. It is a significant factor contributing to preventable diseases and mortality among children, particularly in developing nations [1–3]. Undernutrition, as defined by the World Health Organization (WHO), encompasses wasting, stunting, and being underweight [4]. According to a report by UNICEF in 2022, undernutrition accounts for nearly half of all deaths in children under the age of five. It not only heightens the risk of mortality from common infections but also amplifies the occurrence and severity of such infections, consequently hindering recovery processes [5].

Based on the pooled estimate derived from a meta-analysis, stunting was found to be more prevalent in Sub-Saharan African countries at 33%, followed by underweight at 16%, and wasting at 7%. Specifically, in East Africa, the stunting rate was reported to be 39.0%, surpassing the rates observed in West Africa (31.8%), Southern Africa (30.6%), and Central Africa (28.8%) [6]. Ethiopia has the highest rate of undernutrition among children under the age of five, with stunting affecting 37–42% of the population. Similar to other African countries, stunting is the most prevalent form of undernutrition. Additionally, wasting and underweight rates in Ethiopia were reported to be 7–33% and 1–15%, respectively [7, 8].

The WHO has implemented various strategies to address undernutrition in developing countries. One of the key recommendations is to promote breastfeeding for at least two years or beyond, as breast milk is considered to provide essential nutrition for growth and development, particularly in low-income settings [9]. The first two years of life are crucial for achieving optimal growth and development. Inadequate nutrition during this period can lead to stunting, a condition that may have long-lasting effects on a child’s growth trajectory. Breast milk, being safe and sterile, is rich in antibodies that help protect children from various common childhood illnesses. Prolonged breastfeeding, while beneficial in many contexts, can lead to undernutrition in certain situations [10, 11]. Some studies indicate that children who are breastfed for extended periods may develop a poor appetite and refuse to consume solid foods. This reluctance can stem from an increased attachment to breastfeeding as the child ages, which may overshadow their interest in other food sources [12, 13].

As children continue to breastfeed beyond the age of one, they might become less inclined to explore complementary foods, leading to inadequate intake of essential nutrients. This can result in a reliance on breast milk alone, which may not provide all the necessary nutrients for optimal growth and development, consequently, this can predispose them to malnutrition11]. Research has shown that prolonged breastfeeding can reduce overall food intake. For instance, a study conducted in Pakistan found that children who were breastfed beyond 19 months exhibited higher rates of malnutrition [10].Therefore, this study aimed to investigate the association between breastfeeding duration and undernutrition.

## Methods and materials

### Study area and period

The DHS data was collected from 11 geographic areas, including 9 regions and 2 city administrations in Ethiopia, namely: Tigray, Affar, Amhara, Oromiya, Somali, Benishangul-Gumuz, Southern Nations, Nationalities, and Peoples (SNNP), Gambela, Harari, Addis Ababa, and Dire Dawa. This study utilized the demographic and health survey data conducted in 5 phases between 2000 and 2019 in Ethiopia.

### Data source

The data for this study was extracted from nationally representative Ethiopian DHS surveys conducted between 2000 and 2019. The datasets were obtained through the DHS program and can be accessed at https://dhsprogram.com/customcf/legacy/data/download_dataset.cfm?Filename= AOBR62DT.zip&Tp=1&Ctry_Code=AO&surv_id=395&dm=1&dmode=nm.

Since 2000, the DHS surveys have been conducted every five years, following the standard sampling procedure, data collection, and coding protocols established by the DHS program on an international level [14]. The study utilized the children’s files from the Demographic and Health Surveys (DHS) Program. Permission to access and use the data for the current study was granted by the DHS Program’s data archivist. The EDHS comprises a series of cross-sectional national surveys. Data from the five waves of the Ethiopian Demographic and Health Survey (EDHS) conducted between 2000 and 2019 were utilized in this study. The research was carried out across nine regions and two city administrations (Addis Ababa and Dire Dawa, the country’s two largest metropolitan cities). Data collection was conducted using a stratified two-stage cluster sampling technique [15].

**Fig 1:**
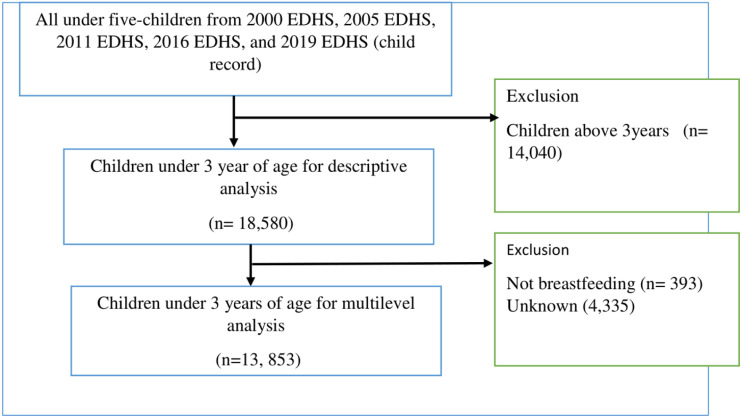
Flow chart for inclusion and exclusion criteria.

For the initial selection stage, all EDHS data from 2000 and 2005 were collected based on the 1994 population and housing census frame, whereas the data from 2011 and 2019 were obtained using the 2007 population and housing census frame (EDHS, 2000, 2005, 2011, 2019). The population list was used to stratify regions into urban and rural areas in the initial stage. Subsequently, each enumeration area or cluster was selected thoughtfully, followed by the selection of households from each enumeration area. The current study encompassed 18,580 children aged 0 to 36 months who participated in one of the four EDHS surveys. The analysis included all participants with complete data for the outcome variable. Children with incomplete height-for-age and weight-for-height records, missing values, and those who were not breastfed were excluded from the study (Fig 1). Upon obtaining permission, the dataset was acquired through the MEASURE DHS platform (https://dhsprogram.com)

### Outcome variable

The outcome variable of this study was undernutrition, defined as comprising underweight, wasting, and stunting. Stunting is characterized by a height-for-age measurement that falls below -2 standard deviations (SD) of the WHO Child Growth Standards median. Wasting is identified when weight-for-height is less than -2 SD of the WHO Child Growth Standards median, while underweight is defined as weight-for-age falling below -2 SD of the same standards [16].

### Explanatory variables

The explanatory variables were chosen based on their reported association with the outcome variable in the literature. Child-level variables included child age, sex preceding birth interval, and having diarrhea, cough, or fever in the two weeks preceding the survey. As household-level variables, the education status of both parents, maternal age, occupation of both parents, and wealth status were considered. Individual-level variables (level-1) in this analysis are the sum of child-level and household-level variables. The variables at the community level (level-2) were residence (urban/rural), source of drinking water (not piped/piped), type of toilet, and region.

### Data analysis

To ensure the survey’s representativeness and to obtain reliable statistical estimates, weighted frequencies and percentages were computed for all variables. Due to the hierarchical nature of the EDHS data, i.e., children were nested in households and households were nested in the community, a multilevel mixed-effects logistic regression analysis was performed using Stata version 14.1 to take into account the clustering effect for the outcome (underweight, wasting and stunting). In bivariate analysis, variables with p-values less than 0.2 were chosen for multivariable analysis. Then, four models were fitted: the empty model (with no independent variables), model II (adjusted for only individual-level variables), model III (adjusted for only community-level variables), and model IV (adjusted for both individual-level and community-level variables simultaneously). Finally, for the best-fitted model, an adjusted odds ratio (AOR) with a 95% confidence interval (CI) was reported. To estimate the goodness of fit of the models, the Akaike information criterion (AIC), the Bayesian information criterion (BIC), and the log-likelihood ratio test were used, with the model with the highest Log likelihood test value and the lowest AIC and BIC values considered to be the best-fit model.

### Random Effect Results

The random-effects model revealed that the prevalence of underweight, stunting, and wasting varies among communities. The ICC of 2.22% for underweight indicates that a small portion of the total variation is attributed to differences between communities. The MOR of 1.038 shows a slight increase in the odds of underweight when comparing children from different communities. The cluster-level variance of 0.0749075 in the final model quantifies the remaining variability in underweight prevalence due to community differences after considering the predictors.

For stunting, the ICC of 2.52% suggests that a small proportion of the total variation is due to community-level differences. The MOR of 1.223 indicates a moderate amount of variability in stunting rates between communities, with higher odds of stunting in different communities. The cluster-level variance of 0.082 in the empty model reflects the variability in stunting attributed to community differences before incorporating predictors.

In the case of wasting, the ICC of 3.31% signifies a small proportion of the total variation arising from differences between communities. The MOR of 0.418 suggests lower odds of stunting when comparing different communities, indicating relatively low variability in stunting rates among communities. The cluster-level variance of 0.0875314 in the empty model represents the unaccounted variability in stunting attributed to differences between communities.

### Ethical Approval and Informed Consent

Ethical approval was granted by the MEASURE Demographic and Health Surveys (DHS) program following the acceptance of a research proposal to utilize its survey datasets. Since this study involved the use of secondary data, participant consent was not require.

## Results

### Socio-demographic characteristics of the respondents and undernutrition

More than half of the children who were underweight (57.57%), wasted (54.89%), or stunted (52.65%) were born to mothers aged 25 to 34. Additionally, over two-thirds of children with mothers lacking formal education experienced undernutrition, with rates of underweight at 78%, wasting at 78%, and stunting at 75%. In terms of partners’ education, 57% of underweight children, 56.7% of stunted children, and 53.5% of wasted children had fathers with no formal education. Undernutrition was prevalent among children whose fathers worked in agriculture, with rates of underweight at 68.9%, wasting at 66.8%, and stunting at 66.9%. Moreover, more than half of the undernourished children had mothers who had given birth within the past two years (Table 1).

**Table 1:** Participants’ characteristics and children’s undernutrition among under 3 years of age in Ethiopia from 2000-2019.

Variable		Underweight		Wasting		Stunting	
	Categories	Weight sample and percentage					
**Maternal and partner-related factors**							
Age		Yes (%)	No (%)	Yes (%)	No (%)	Yes (%)	No (%)
	15-24	1,438(25.55)	3,691(28.49)	706(27.45)	4,423(27.63)	1,229(20.39)	3,571(45.64)
	25-34	3,240(57.57)	7,319(56.50)	1,414(54.98)	9,145(57.12)	3,174(52.65)	3,316(42.38)
	35-46	950(16.88)	1,944(15.01)	452(17.57)	2,442(15.25)	1,625(26.96)	938(11.99)
Employment	Unemployed	2,530(44.95)	5,806(44.82)	1,233(47.94)	7,103(44.37)	3,469(43.56)	4,867(45.83)
	Employed	2,497(44.37)	4,982(38.46)	1,083(42.11)	6,396(39.95)	3,572(44.86)	3,907(36.79)
	Unknown	601(10.68)	2,166(16.72)	256(9.95)	2,511(15.68)	922(11.58)	1,845(17.37)
Educational status	No formal education	4,397(78.13)	8,177(63.12)	2,004(77.92)	10,570(66.02)	5,980(75.10)	6,594(62.10)
	Primary education	1,054(18.73)	3,296(25.44)	476(18.51)	3,874(24.20)	1,645(20.66)	2,705(25.47)
	Secondary and above	177(3.14)	1,481(11.43)	92(3.58)	1,566(9.78)	338(4.24)	1,320(12.43)
Husband’s education	No formal education	3,215(57.13)	5,168(39.90)	1,457(56.65)	6,926(43.26)	4,257(53.46)	**4,126(38.85)**
	Primary education	1,355(24.08)	3,477(26.84 0	585(22.74)	4,247(26.53)	2,042(25.64)	2,790(26.27)
	Secondary and above	380(6.75)	1,905(14.71	228(8.86)	2,057(12.85)	621(7.80)	1,664(15.67)
	unknown	678(12.05)	2,404(18.56)	302(11.74)	2,780(17.36)	1,043(13.10)	2,039(19.20)
Husband’s job	not working	158(2.81)	443(3.42)	95(3.69)	506(3.16)	200(2.51)	401(3.78)
	Agriculture	3,878(68.91)	6,840(52.80)	1,709(66.45)	9,009(56.27)	5,328 (66.91)	5,390(50.76)
	Business and others	950(16.88)	3,350(25.86)	479(18.62)	3,821 (23.87)	1,442(18.11)	2,858(26.91)
	unknown	642(11.41)	2,321(17.92)	289(11.24)	2,674(16.70) 95	993(12.47)	1,970(18.55)
Marriage status	Never married	358(6.36)	831(6.42)	169(6.57)	1,020(6.37)	539(6.77)	650(6.12)
	Married	5,270(93.64)	12,123(93.58)	2,403(93.43)	14,990 (93.63)	7,424(93.23)	9,969(93.88)
Birth Space (14,912)	≤2	2,770(58.43)	5,240(51.52)	1,214(55.89)	6,796(53.34)	3,797(58.12)	4,213(50.28)
	>2	1,971(41.57)	4,931(48.48)	958(44.11)	5,944(46.66)	2,736(41.88)	4,166(49.72)
**Child related factors**							
Sex	Male	3,089(54.89)	6,332(48.88)	1,458(56.69)	7,963(49.74)	4,270(53.62)	5,151(48.51)
	Female	2,539(45.11)	6,622(51.12)	1,114(43.31)	8,047(50.26)	3,693 (46.38)	5,468(51.49)
Age of the child	<12	1,001(17.79)	3,426(26.45)	806(31.34 |)	3,621(22.62)	990(12.43)	3,437(32.37)
	13-24	2,182(38.77)	4,833(37.31)	1,025(39.85)	5,990(37.41)	3,182(39.96)	3,833 (36.10)
	25-3	2,445(43.44)	4,695 (36.24)	741(28.81)	6,399 (39.97)	3,791(47.61)	3,349(31.54)
Fever past two weeks	Yes	1,574(27.97)	2,487(19.20)	817(31.77)	3,244(20.26)	1,929(24.22)	2,132(20.08)
	No	4,054(72.03)	10,467(80.80)	1,755(68.23)	12,766(79.74)	6,034(75.78)	8,487(79.92)
Duration of breastfeeding (13,853)	1^st^ year of life	1,142(26.16)	3,658(38.55)	841(40.69)	3,959(33.59)	1,229(20.39)	3,571(45.64)
	2^nd^ years of life	2,149(49.23)	4,341(45.75)	943(45.62)	5,547(47.06)	3,174(52.65)	3,316(42.38)
	3^rd^ years of life	1,074(24.60)	1,489(15.69)	283(13.69)	2,280(19.34)	1,625(26.96)	938(11.99)
Recent diarrhea	Yes	1,468(26.08)	2,228 (17.20)	737(28.65)	2,959(18.48)	1,800(22.60)	1,896(17.85)
	No	566(10.06)	8,648(66.76)	1,595(62.01)	10,647(66.50)	5,295(66.50)	6,947(65.42)
	Unknown	566(10.06)	2,078(16.04)	240(9.33)	2,404(15.02)	868(10.90)	**1,776(16.72)**
**Household factors**							
Wealth index	Poorest	1,548(27.51)	2,879(22.22)	790(30.72)	3,637(22.72)	1,968(24.71)	2,459(23.16)
	Poorer	777(13.81)	1,740(13.43)	349(13.57)	2,168(13.54)	1,114(13.99)	1,403(13.21)
	Middle	600(10.66)	1,606(12.40)	280(10.89)	1,926(12.03)	919(11.54)	1,287(12.12)
	Richer	465(8.26)	1,569(12.11)	198(7.70)	1,836(11.47)	796(10.00)	1,238(11.66)
	Richest	398(7.07)	2,484(19.18)	201(7.81)	2,681(16.75)	697(8.75)	2,185(20.58)
	Unknown	1,840(32.69)	2,676(20.66)	754(29.32)	3,762(23.50)	2,469(31.01)	2,047(19.28)
weakly TV used	Yes	153(2.72)	1,271(9.81)	75(2.92)	1,349(8.43)	328(4.12)	1,096(10.32)
	No	4,905(87.15)	9,607(74.16)	2,257(87.75)	12,255(76.55)	6,761(84.91)	7,751(72.99)
	Unknown	570(10.13)	2,076(16.03)	240(9.33)	2,406(15.03)	874(10.98)	1,772(16.69)
r weakly radio used	Yes	1,234(21.93)	4,203(32.45)	565(21.97)	4,872(30.43)	1,961(24.63)	3,476(32.73)
	No	4,290(76.23)	8,554(66.03)	1,964(76.36)	10,880(67.96)	5,854(73.52)	6,990(65.83)
	Unknown	104(1.85)	1967(1.52)	43(1.67)	258(1.62)	148(1.86)	153(1.43)
Water source	non-piped	4,910(87.26)	10,085(77.85)	2,221(86.35)	12,774(79.79)	6,853(86.07)	8,142(76.67)
	Piped	717(12.74)	2,869(22.15)	351(13.65)	3,235(20.21)	1,109(13.93 |)	2,477(23.33)
Toilet facility	VIP	547(9.72)	2,662(20.55)	270(10.50)	2,939 (18.36)		2,271(21.39)
	Un improved sanitation	1,152(20.47)	3,647 (28.16)	495(19.25)	4,304(26.89)		2,961(27.89)
	Open defecation	3,929(69.81)	6,643(51.29)	1,807(70.26)	8,765(54.75)		5,385 (50.72)
**Community-related factors**							
Settlement	Urban	528(9.38)	2,809(21.68)	287(11.16)	3,050(19.05)	898(11.28)	2,439(22.97)
	Rural	5,100(90.62)	10,145(78.32)	2,285(88.84)	12,960(80.95)	7,065(88.72)	8,180(77.03)

### Child related Characteristics and Undernutrition among Children under 3 years of age

Children who were breastfed until their second and third year of life exhibited a higher prevalence of undernutrition. Among those breastfed until their second year, more than half (52.7%) were stunted, 49% were underweight, and 45.6% were wasted. Similarly, 26% of children breastfed until their third year experienced stunting, and 24% were underweight. Undernutrition was also more common among children who had diarrhea within the past two weeks, with over one-fourth of underweight children (26.6%) and wasting children (28.75%) reporting recent episodes of diarrhea (Table 1).

### Household characteristics undernutrition among children under 3 years of age in Ethiopia from 2000–2019

Children from poorer families exhibited higher levels of undernutrition. Twenty-eight percent of underweight children, 30.7% of wasting children, and 24.7% of stunted children were from poorer households. The majority of children affected by undernutrition resided in homes without access to piped water, lacked television or radio, and practiced open-field defecation. Additionally, nearly all children experiencing undernutrition were from rural (Table 1).

### Prevalence of undernutrition among under Children 3 years of age in Ethiopia from 2000–2019

**Fig 2:**
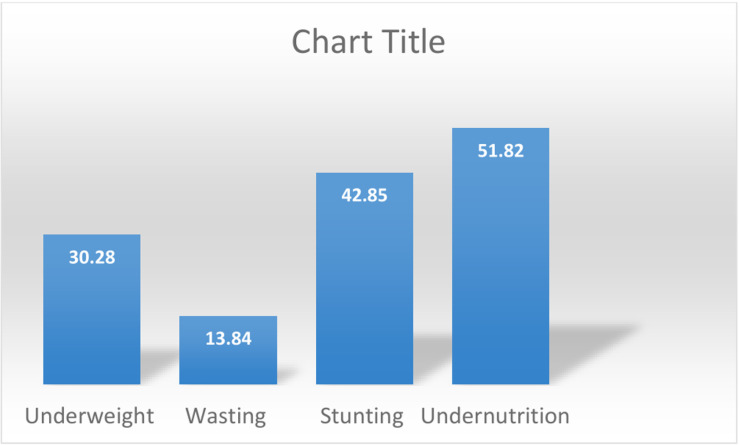
Prevalence of undernutrition among 0-3 years of children.

The overall prevalence of undernutrition among children under 3 years of age was 51.8% (95% CI: 51.10, 52.54). Specifically, the prevalence of stunting was 42.9% (95% CI: 42.14, 43.56), followed by underweight at 30.28% (95% CI: 29.63, 30.92), and wasting at 13.84% (95% CI: 13.35, 12.04) (Fig 2).

### Factors Associated with Undernutrition among under 3 years of age in Ethiopia from 2000-2019

In the bivariate analysis of children’s undernutrition, various factors were evaluated for inclusion in multivariable logistic regression, including maternal and partner-related aspects, child-related variables, household factors, and community-related factors. After adjusting for potential confounders, children breastfed into their second year had a higher likelihood of being underweight (AOR: 1.55; 95% CI: 1.41, 1.70), while those breastfed into their third year had an even greater risk (AOR: 2.28; 95% CI: 2.03, 2.56). Additionally, maternal secondary education (AOR: 0.61; 95% CI: 0.47, 0.78), husband’s education, birth spacing greater than two years (AOR: 0.82; 95% CI: 0.75, 0.90), recent child fever (AOR: 1.27; 95% CI: 1.156, 1.39), recent diarrhea (AOR: 1.34; 95% CI: 1.22, 1.48), unimproved sanitation (AOR: 1.21; 95% CI: 1.02, 1.44), and open defecation (AOR: 1.55; 95% CI: 1.33, 1.80) were significantly associated with underweight. For stunting, breastfeeding during the second year increased risk (AOR: 2.91; 95% CI: 2.66, 3.19) and even more so in the third year (AOR: 5.45; 95% CI: 4.83, 6.15). Conversely, the likelihood of wasting decreased among children breastfed during these years (AOR: 0.75; 95% CI: 0.67, 0.83 for the second year and AOR: 0.50; 95% CI: 0.43, 0.60 for the third year; Tables 1–4).

**Table 2:** Multivariable Multilevel Mixed Effect Logistic Regression Model Result of Underweight among Children under 3 years of age in Ethiopia from 2000-2019.

		Underweight			
Variable	Categories	Model_1	Model_2	Model_3	Model_3
**Individual-level variables**					
Age			AOR (95%CI)	AOR (95%CI)	AOR (95%CI)
	15-24		1		1
	25-34		1.09(0.97,1.22)		1.09(0.97,1.22)
	35-46		1.00(0.87, 1.15)		1.00(0.87, 1.15)
Employment	Unemployed		1		
	Employed		1.02(0.93,1.11)		1.02(0.93, 1.11)
Educational status	No formal education		1		1
	Primary education		0.91(0.81,1.02)		0.91(0.81,1.02)
	Secondary and above		0.59(0.46,0.76)		0.61 (0.47, 0. 78)^*^
Husband’s education	No formal education		1		1
	Primary education		0.76(0.69,0.84)		0.77(0.69,0.84)^*^
	Secondary and above		0.58(0.48,0.69)		0.60(0.50, 0.71)^*^
Husband’s Employment	not working		1		1
	Agriculture		1.26(0.98,1.63)		1.22(0.95, 1.58)
	Business and others		1.07(0.82,1.41)		1.12(0.86, 1.48)
Marriage status	Never married		1		1
	Married		1.34(1.22,1.47)		0.97(0.81,1.17)
Birth Space (14,912)	≤2		1		1
	>2		0.83(0.75,0.90)		0.82(0.75, 0.90)^*^
Fever past two weeks	No		1		1
	Yes		1.27(1.16,1.40		1.27(1.156, 1.39)^*^
**Duration of breastfeeding**	**1**^**st**^ **year of life**		**1**		1
	**2**^**nd**^ **years of life**		**1.55(1.41,1.70)**		**1.55(1.41, 1.70)** ^**^
	**3**^**rd**^ **years of life**		**2.28(2.03,2.56)**		**2.28(2.03,2.56)** ^**^
Recent diarrhea	No		1		1
	Yes		1.34(1.22, 1.47)		1.34(1.22, 1.48)^*^
Wealth index	Poorest		1		1
	Poorer		0.84(0.72,0.97)		0.83(0.72,0.96)^*^
	Middle		0.71(0.61,0.84)		0.71(0.61,0.83)^*^
	Richer		0.60(0.51,0.71)		0.60(0.50,0.71)^*^
	Richest		0.64(0.52,0.76)		0.70(0.82,0.87)^*^
weakly TV used	No		1		1
	Yes		0.55(0.43,0.70)		0.55(0.43,0.74)^*^
**Community level**					
Water source	non-piped			1	1
	Piped			0.93(0.83 1.04)	1.12(0.97, 1.29)
Toilet facility	VIP			1	
	Un improved sanitation			1.15(1.02, 0.31)	1.21(1.02, 1.44)^*^
	Open defecation			2.08(1.86, 2.35)	1.55(1.33,1.80)^*^
Settlement	Urban			1	1
	Rural			1.87(1.64,2.14)	1.31(1.07, 1.61)^*^
Parameters		Null model (model-0)	Modela I	Modela II	Model IV (final modela
Cluster level variance (SE)		0.1210807	0.0724947	0.1054758	0.0749075
PCV (%)		ref	52.2%	22.2%	55.4%
ICC or VPC (%)		0.35%	0.22%	0.31%	0.22%
MOR		**1.062**	1.037	**1.054**.	**1.038**.
Fit criteria (model diagnostics)					
Loglikelihood (LL)		-11338.574	-7111.0343	-10884.489	-7101.3081
AIC		22681.15	14290.07	21786.98	14276.62
BIC		**22696.81**	14541.68	21857.45	14550.42

**Table 3:** Multivariable Multilevel Mixed Effect Logistic Regression Model Result of Stunting among Children under 3 years of age in Ethiopia from 2000-2019.

		Stunting			
Variable	Categories	Model_1	Model_2	Model_3	Model_3
**Individual-level variables**					
Age			AOR (95%CI)	AOR (95%CI)	AOR (95%CI)
	15-24		1		1
	25-34		1.02(0.92,1.14)		1.03(0.93,16)
	35-46		0.92(0.80, 1.05)		0.93(0.80, 1.06)
Employment	Unemployed		1		
	Employed		1.07(0.98,1.16)		1.06(0.98,1.16)
Educational status	No formal education		1		1
	Primary education		0.89(0.80,0.99)		0.90(0.80,1.00)
	Secondary and above		0.57(0.46,0.71)		0.60 (0.48 0. 75)^*^
Husband’s education	No formal education		1		1
	Primary education		0.82(0.75,0.90)		0.84(0.76,0.92)^*^
	Secondary and above		0.60(0.51,0.70)		0.63(0.53, 0.73)^*^
Husband’s Employment	not working		1		1
	Agriculture		1.5(10.17,1.92)		1.48(1.56, 1.89)^*^
	Business and others		1.24(0.96,1.61)		1.30(1.01, 1.69)^*^
Marriage status	Never married		1		1
	Married		0.86(1.22,1.02)		0.86(0.71,1.03)
Birth Space (14,912)	≤2		1		1
	>2		0.77(0.70,0.84)		0.77(0.71, 0.84)^*^
Fever past two weeks	No		1		1
	Yes		1.03(0.94,1.13)		1.02(0.93, 1.12)
**Duration of breastfeeding**	**1**^**st**^ **year of life**		**1**		1
	**2**^**nd**^ **years of life**		**2.90(2.64, 3.17)**		**2.91(2.66, 3.19)** ^**^
	**3**^**rd**^ **years of life**		**5.50(4,83,6.20)**		**5.45(4.83,6.15)** ^**^
Recent diarrhea	No		1		1
	Yes		1.16(1.06, 1.28)		1.17(1.06, 1.29)^*^
Wealth index	Poorest		1		1
	Poorer		0.93(0.81,1.07)		0.98(0.85,1.14)
	Middle		0.88(0.75,1.07)		0.95(0.81,1.12)
	Richer		0.81(0.69,0.95)		0.90(0.76,1.06)
	Richest		0.73(0.61,0.88)		0.84(0.69,1.04)
weakly TV used	No		1		1
	Yes		0.70(0.57,0.86)		0.75(0.61,0.92)^*^
**Community level**					
Water source	non-piped			1	1
	Piped			0.93(0.83 1.04)	0.96(0.84, 1.10)
Toilet facility	VIP			1	
	Un improved sanitation			1.15(1.02, 0.31)	1.41(0.97, 1.33)
	Open defecation			2.08(1.86, 2.35)	1.37(1.19,1.57)^*^
Settlement	Urban			1	1
	Rural			1.87(1.64,2.14)	1.02(0.84 1.24)
Parameters		Null model (model-0)	Model I	Model II	Model IV (final model)
Cluster level variance (SE)		0..0853843	0.0856112	0.0769911	0.0821
PCV (%)		ref	14.00	3.25%	14.04
ICC or VPC (%)		2.53%	2.54%	2.29%	2.52
MOR		1.128	1.129.	1.114.	1.123
Fit criteria (model diagnostics)					
Loglikelihood (LL)		-12649.183	-7108.6755	-12420.692	-7400.0932
AIC		25302.37	14874.9	24851.38	14874.19
BIC		25318.03	15126.51	24890.53	15147.99

**Table 4:** Multivariable Multilevel Mixed Effect Logistic Regression Model Result of Wasting among Children under 3 years of age in Ethiopia from 2000-2019.

		Wasting			
Variable	Categories	Model_1	Model_2	Model_3	Model_3
**Individual-level variables**					
Age			AOR (95%CI)	AOR (95%CI)	AOR (95%CI)
	15-24		1		1
	25-34		0.97(0.84, 1.11)		0.96(0.85, 1.12)
	35-46		1.04(0.88, 1.12)		0.89(0.73, 1.11)
Employment	Unemployed		1		1
	Employed		1.03(0.93,1.15)		1.04(0.94,1.16)
Educational status	No formal education		1		1
	Primary education		0.91(0.78,1.05)		0.91(0.79,1.06)
	Secondary and above		0.58(0.42,1.80)		0.60(0.44,1.83)
Husband’s education	No formal education		1		1
	Primary education		0.76(0.67,0.86)		0.77(0.68,0.88)^*^
	Secondary and above		0.87(0.70, 1.07)		0.90(0.73, 1.10)
Husband’s Employment	not working		1		1
	Agriculture		0.88(0.66, 1.18)		0.87(0.65, 1.17)
	Business and others		0.80(0.59, 1.09)		0.81(0.59, 1.11)
Marriage status	Never married		1		1
	Married		0.90(0.71,1.13)		0.90(0.72,1.13)
Birth Space (14,912)	≤2		1		1
	>2		0.95(0.85, 1.06)		0.96(0.86, 1.07)
Fever past two weeks	No		1		1
	Yes		1.43(1.28, 1.60)		1.41(1.26, 1.59)^*^
**Duration of breastfeeding**	**1**^**st**^ **year of life**		1		1
	**2**^**nd**^ **years of life**		**0.75(0.67, 0.83)**		**0.75(0.67, 0.83)** ^**^
	**3**^**rd**^ **years of life**		**0.52(0.44,0.61)**		**0.50(0.43,0.60)** ^**^
Recent diarrhea	No		1		1
	Yes		1.35(1.20, 1.52)		1.35(1.20, 1.52)^*^
Wealth index	Poorest		1		1
	Poorer		0.82(0.69,0.98)		0.87(0.72,1.03)
	Middle		0.69(0.57,0.84)		0.76(0.62,0.93)^*^
	Richer		0.52(0.43,0.66)		0.59(0.47,0.74)^*^
	Richest		0.57(0.45,0.73)		0.63(0.48,0.83)^*^
weakly TV used	No		1		1
	Yes		0.71(0.53,0.97)		0.74(0.54,1.00)
**Community level**					
Water source	non-piped			1	1
	Piped			)0.92(0.79, 106)	1.07(0.91, 1.30)
Toilet facility	VIP			1	
	Un improved sanitation			1.08 (0.91, 1.29)	1.44(0.97, 1.43)
	Open defecation			1.90(1.62, 2.23)	1.43(1.98,2.19)^*^
Settlement	Urban			1	1
	Rural			1.33(1.12,1.59)	0.96(0.74 1.25)
Parameters		Null model (model-0)	Model I	Model II	Model IV (final model
Cluster level variance (SE)		0.1251949	0.0817521	0.12509	0.0875314
PCV (%)		ref	3.37%	1.30	3.31%
ICC or VPC (%)		3.67%	2.42%	3.67%	2.43%
MOR		1.194	1.122	1.194	0.418
Fit criteria (model diagnostics)					
Loglikelihood (LL)		-7448.4201	-5023.0331	-7393.4932	-5022.1528
AIC		14900.84	10112.07	14796.99	10116.31
BIC		14916.5	10356.24	14836.14	10382.67

## Discussion

This research aimed to establish the link between the length of breastfeeding and undernutrition in Ethiopia. The findings reveal that there is a notable prevalence of undernutrition in children breastfed until the age of three. The most common issue among these children was stunting (45.6%), followed by underweight (31.5%). This aligns with a similar study conducted in Pakistan, which reported stunting at 40.6% and underweight at 33.9% among children who were on breast feeding until age three [10]. The reason for this might be the inability to access sufficient and varied food at the right developmental stage. A study in Ghana suggests that extended breastfeeding could restrict overall food consumption, potentially contributing to malnutrition [17]. Complementary foods must be nutritionally adequate, safe, and appropriately fed to meet the energy and nutrient demands of the young infant. However, complementary feeding is frequently riddled with issues, such as foods being too dilute, not being provided frequently enough or in insufficient amounts, or replacing breast milk with lower quality [9].

The research revealed a significant association between the length of breastfeeding and stunting. Children breastfed until two years old were 2.9 times more likely to experience stunting, and those breastfed until three were 5.45 times more likely. This is consistent with data from Nineteen Demographic and Health Surveys in Sub-Saharan Africa (SSA), indicating that children breastfed beyond the first year were notably shorter and lighter than those who were not breastfed [12]. Similarly, a recent study demonstrated that the length of breastfeeding significantly influenced child development. Children breastfed beyond the first year had a 3-6 times higher likelihood of experiencing severe stunting [10]. This could be due to infants who show less interest in complementary foods initially choosing to breastfeed for longer periods, thus rejecting other foods and causing nutrient deficiencies and impaired growth. Furthermore, prolonged breastfeeding may have a negative impact on infant appetite and growth [18].

In this study, being underweight was found to have a strong association with the duration of breastfeeding. The chances of a child being underweight increased by 55% if they were breastfed until their second year, and by 128% if breastfed until their third year. This aligns with a study in Guinea-Bissau showing that children with low weight for their age were breastfed for an extended period compared to other children [19]. Similarly, a study from Ghana indicated that continued nursing after the age of 18 months is related to an increased risk of malnutrition [20]. However, the results of this study indicated that the duration of breastfeeding played a protective role against wasting. The probability of wasting decreased by 50% in children breastfed until their third year, and by 25% in children breastfed until their second year. A study from Pakistan also reported similar results, showing lower rates of wasting in children breastfed until their third year [10].

## Conclusion

The prevalence of undernutrition was significant among children who breastfed for more than one year, and breastfeeding duration was inversely associated with stunting and underweight. This finding can be utilized as a starting point for future longitudinal studies to determine the effect of breastfeeding length on undernutrition. It is therefore recommended that a large-scale study be conducted in various countries to assist the World Health Organization in revising breastfeeding guidelines.

## Theoretical and practical implications of the study

The study highlights the critical relationship between breastfeeding duration and undernutrition, reinforcing theoretical frameworks that emphasize early childhood nutrition as vital for growth and development. It underscores the World Health Organization’s recommendation for breastfeeding up to two years, illustrating how prolonged breastfeeding can lead to increased risks of stunting and underweight, particularly when it delays the introduction of complementary foods. Practically, these findings suggest that health programs should focus on educating mothers about the importance of transitioning to a diverse diet after the first year of breastfeeding to mitigate risks associated with prolonged breastfeeding. This could lead to improved child health outcomes by ensuring that children receive adequate nutrition essential for their growth and development.

## Limitations and strategies for minimization

The study has several limitations, including potential recall bias in self-reported breastfeeding duration and other child-related factors, as well as the cross-sectional design that may limit causal inferences. To address these limitations, future research should consider longitudinal designs to track changes over time and implement objective measures for assessing nutritional status. Additionally, enhancing training for data collectors can improve the accuracy of reported information. By addressing these limitations, the reliability of findings can be strengthened, allowing for more effective interventions targeting child nutrition.
